# Transcriptome Mechanisms of Tomato Seedlings Induced by Low-Red to Far-Red Light Ratio under Calcium Nitrate Stress

**DOI:** 10.3390/ijms24043738

**Published:** 2023-02-13

**Authors:** Xiaoting Zhou, Jia Huang, Yirong Gan, Zelin Li, Lihong Su, Zhongqun He, Junwei Yang, Zhihui Wang, Chengyao Jiang, Zhi Huang, Wei Lu, Wangang Zheng

**Affiliations:** 1 College of Horticulture, Sichuan Agricultural University, Chengdu 611130, China; 2 Nanchong Academy of Agricultural Sciences, Nanchong 637002, China; 3 College of Horticulture, China Agricultural University, Beijing 100193, China

**Keywords:** tomato, saline stress, transcriptomic analysis, red light to far-red light ratio, WGCNA

## Abstract

In recent times, the excessive accumulation of nitrate has been one of the main reasons for the secondary salinization of greenhouse soils. Light plays a key role in a plant’s growth, development, and response to stress. A low-red to far-red (R:FR) light ratio could enhance plant salinity tolerance, but the mechanism at a molecular level is unclear. Thus, we analyzed the transcriptome responses of tomato seedlings to calcium nitrate stress under either a low R:FR ratio (0.7) or normal light conditions. Under calcium nitrate stress, a low R:FR ratio enhanced both the antioxidant defense system and the rapid physiological accumulation of proline in tomato leaves, which promoted plant adaptability. Using weighted gene co-expression network analysis (WGCNA), three modules including 368 differentially expressed genes (DEGs) were determined to be significantly associated with these plant traits. Functional annotations showed that the responses of these DEGs to a low R:FR ratio under excessive nitrate stress were enriched in the areas of hormone signal transduction, amino acid biosynthesis, sulfide metabolism, and oxidoreductase activity. Furthermore, we identified important novel hub genes encoding certain proteins, including FBNs, SULTRs, and GATA-like transcription factor, which may play a vital role in low R:FR light-induced salt responses. These findings offer a new perspective on the mechanisms and environmental implications behind low R:FR ratio light-modulated tomato saline tolerance.

## 1. Introduction

The secondary salinization of soil has become the main limiting factor of the sustainable development of agricultural production [1]. However, in contrast to open-field cultivation, the excessive accumulation of Ca(NO_3_)_2_ in secondary saline soil is more prevalent compared to that of NaCl, due to excessive fertilization and intensive farming in greenhouses [2,3]. This excess Ca(NO_3_)_2_ inhibits plant growth and causes ion imbalances, resulting in oxidative stress and reduced water availability [4]. To respond to salinity stress, plants invoke complex defensive mechanisms via physiological and biochemical changes [5]. The plant tissues accumulate high levels of soluble sugars, amino acids, glycine betaine, and proline intracellularly, as a means to cope with high salinity stress [6]. Moreover, reactive oxygen species (ROS) production and scavenging mechanisms, such as those involving superoxide dismutase (SOD), catalase (CAT), and peroxidase (POD), play a critical role in stress physiology [7,8].

A light-emitting diode (LED), as a new lighting source, has a series of advantages, such as high efficiency, energy saving, long life, and adjustability properties. In particular, the proportions of different light qualities can be adjusted.

In vegetable crops, the ratio between red and far-red light (R:FR) determines growth and development, physiological metabolism, yield, and quality. Zhen and Iersel [9] found that reducing the R:FR ratio can enhance the net photosynthetic rate and the actual photochemical efficiency of PSIIin lettuce leaves, thus effectively improving photosynthesis. It has also been found that exposing soybean leaves to low R:FR light produces higher net photosynthetic rates [10]. In addition, the ratio of R:FR affects the photomorphogenesis of plants, and alleviates the damage to plants caused by salt [11] and cold tolerance [12]. Previous research by our team found that plants grown under low R:FR ratios showed alleviated growth inhibition [3]; in pakchoi [13] under calcium nitrate stress, it effectively improved the photosynthetic efficiency. However, the molecular mechanism of low R:FR in improving the salt resistance of tomato plants is still unclear.

Tomato (*Solanum lycopersicum* L.) is a moderately salt-sensitive crop which is widely planted globally due to its high value [14]. This study identified key genes and pathways associated with low R:FR light-treated tomato seedling responses to calcium nitrate stress. Additionally, we used a weighted gene co-expression network analysis (WGCNA) to identify the modules of co-expressed and hub genes related to the salt tolerance network, in addition to physiological response evaluations and an RNA-seq analysis. The purpose of this study was to develop a better understanding of how light regulates salt tolerance at the genetic level, as well as to identify the key regulators and genes that can be manipulated in order to enhance stress tolerance in tomato seedlings.

## 2. Results

### 2.1. Response to Calcium Nitrate Stress

**Figure 1:**
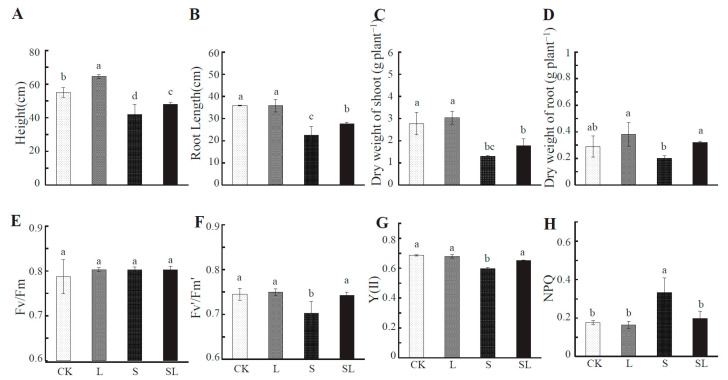
Tomato seedlings plant height (**A**), root length (**B**), dry weight of shoot (**C**), and dry weight of root (**D**) Fv/Fm (**E**), Fv’/Fm’ (**F**), Y(II) (**G**), NPQ (**H**), under different treatments. CK = control, L = + FR, S = + Ca(NO_3_)_2_, SL = + FR + Ca(NO_3_)_2_. Data represent the mean (3 plants per sample, three samples per treatment) ± standard error of the mean. The means for each treatment without common letters were significantly different at *p* ≤ 0.05, according to Duncan’s multiple range test.

According to Figure 1A–D, a low R:FR retio under no stress treatment (L) resulted in increased plant height, but the roots and shoots of weight did not significantly respond. The growth of the tomato plant was significantly inhibited by calcium nitrate stress. The plant height, root length, and dry weight of the shoots and roots of the tomato plant under calcium nitrate stress (S treatment) were significantly reduced—by 23.68%, 36.99%, 53.43%, and 31.03%, respectively. Compared with the S treatment, the plant height (Figure 1A), root length, aboveground dry weight, and underground dry weight of tomato plants under the SL treatment increased significantly—by 14.20%, 22.43%, 37.98%, and 60%, respectively. According to the results shown in Figure 1E, there was no significant change in the maximum quantum efficiency (Fv/Fm) of the PSII photochemistry of tomato leaves. Under calcium nitrate stress, the effective photon yield (Fv’/Fm’) of PSII and the actual electron transport efficiency of PSII (Y(II)) in tomato leaves were significantly decreased (Figure 1F–G), while non-photochemical quenching (NPQ) was significantly increased (Figure 1H). This indicated that calcium nitrate stress significantly inhibited the photoelectric conversion, transmission efficiency, and activity related to the PS II reaction center in tomato seedling leaves. When treated with a low R:FR ratio, the energy and efficiency of the PS II reaction center in the leaves was significantly increased under the SL treatment. Compared with the S treatment, under the SL treatment, the Fv’/Fm’ and Y(II) increased by 9.20% and 5.69%, respectively, and the NPQ decreased by 40.66%.

**Figure 2:**
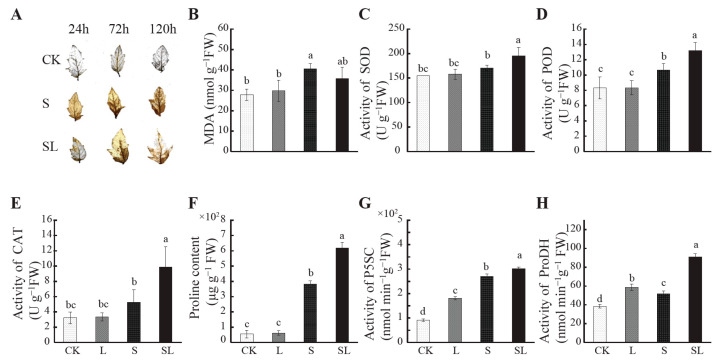
Tomato seedlings’ histochemical staining of H_2_O_2_ (**A**), MDA content (**B**), activity of SOD (**C**), activity of POD (**D**), activity of CAT (**E**), proline content (**F**), activity of P5CS (**G**), and activity of ProDH (**H**) under different treatments. CK = control, L = + FR, S = + Ca(NO_3_)_2_, SL = + FR + Ca(NO_3_)_2_. Data represent the mean (3 plants per sample, three samples per treatment) ± standard error of the mean. The means for each treatment without common letters were significantly different at *p* ≤ 0.05 according to Duncan’s multiple range test.

In order to observe the change of H_2_O_2_ content in tomato leaves, we detected the content of H_2_O_2_ using the diaminobenzidine (DAB) method. Under salt stress, tomato leaves with a low R:FR ratio exhibited significantly lower levels of H_2_O_2_ (Figure 2A). Malondialdehyde (MDA) accumulates in tomato leaves when membrane-permeable lipid peroxidation is performed. Calcium nitrate stress for 5d led to an increase in MDA in tomato leaves, while there was a significant reduction in MDA in the SL treatment when compared with S (Figure 2B). Calcium nitrate stress for 5d increased the activities of SOD, POD, and CAT in the tomato leaves, and the activities of POD significantly increased by 28.55%. Compared with S, SOD, POD, and CAT in the SL treatment increased by 14.21%, 23.71%, and 88%, respectively (Figure 2C–E). Under calcium nitrate stress, leaf antioxidant enzyme activities increased significantly with a low R:FR ratio.

At the same time, the proline content in the leaves of the tomato seedlings increased by 596.43% under the S treatment. The highest proline content was observed in SL, which increased by 62.63%, when compared with S (Figure 2F). P5CS is the key enzyme for proline synthesis. The activity of P5CS in tomato seedling leaves was significantly increased (198.06%) under calcium nitrate stress, and the activity of P5CS was further increased by 11.48% in SL when compared with S. The change trend of proline dehydrogenase (ProDH) under calcium nitrate stress was consistent with that of P5CS, with the highest activity of ProDH observed in SL (Figure 2G–H). According to Figure 2, under the L treatment, there was no difference, except that the activities of P5CS and ProDH increased by 98.88% and 52.43%, respectively.

### 2.2. Sequencing and Transcriptome Assembly

To further examine the key genes involved in regulating tomato seedling salinity tolerance, a transcriptome analysis of leaves under calcium nitrate stress and well-watered conditions was conducted. Through pairwise comparisons, 6802 DEGs were identified.

**Figure 3:**
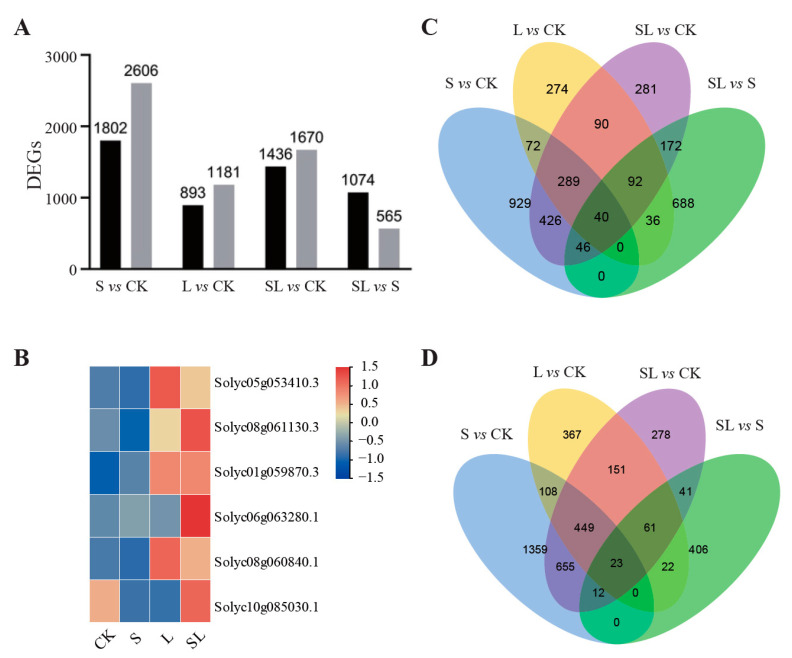
Transcriptome analysis of tomato leaves following low R:FR ratio light treatment and/or calcium nitrate stress. (**A**) DEGs expression variation among different treatments in tomato leaves. (**B**) Expression analysis of genes involved in red and far-red light signal transduction among different treatments; red and blue color indicate up-regulated and down-regulated gene expression, respectively. (**C**,**D**) Venn diagram for the expression variation among different treatments in tomato leaves from up-regulated DEGs, down-regulated DEGs, respectively.

From the results, comparisons between S and CK, L and CK, SL and CK, and SL and S produced 4408, 2074, 3106, and 1639 DEGs, respectively. According to Figure 3A, 1074 DEGs were upregulated in SL compared to S, while 565 DEGs were downregulated. In addition, the hierarchical clustering analysis provided an overview of the DEG expression patterns (Figure S1).

In order to categorize the DEGs, a GO enrichment analysis was conducted (Figure 3B, Figure S2). Among the GO enrichment pathways of SL vs. S, the far-red light related pathways were significantly enriched. The five genes in GO: 0,010,017 (red or far-red light signaling pathway) and GO: 0,071,489 (cellular response to red or far-red light) were all upregulated in SL. Among them, solyc08g061130.3 (HY5) and solyc01g059870.3 (phyB) were significantly enriched in SL and L, indicating that far-red light treatment affected the expression of HY5 and phyB related genes (Figure 3B).

From Figure S2, the comparison of S and CK showed that small molecule biosynthesis, organic acid biosynthesis, carboxylic acid biosynthesis, and lipid biosynthesis are significantly more enriched GO terms. In the molecular functional category, the transmembrane transport activity, ionic transmembrane transporter activity, transferase activity, enzyme activity inhibitor, and anionic transmembrane transporter activity of inorganic molecular entities were significantly enriched. A comparison of SL vs. S revealed that the significantly enriched GO terms were lipid biosynthesis, oxidoreductase activity, acting on paired donors, with incorporation or reduction in molecular oxygen, extracellular region, and ribosome subunit. In SL and CK, the transmembrane transport activity of inorganic molecules was the most significantly enriched GO term. In the comparison between L and CK, protein chromophore linkage and protein dimerization activity were the most significantly enriched GO terms.

Further exploration of DEG biological functions was performed using KEGG analyses (Figure S3). In each comparison, the highest enrichment levels were found in ten pathways in plants. As for the KEGG analyses in the comparison of S vs. CK, we found that the number of genes in carbon metabolism, amino acid biosynthesis, MAPK signaling pathway, glycolysis, and sugar metabolism synthesis accounted for a large proportion, which may be a significant contributor to calcium nitrate stress. In contrast, the top five significantly enriched pathways in SL compared to S were ribosome, phenylpropanoid biosynthesis, plant hormone signal transduction, steroid biosynthesis, and flavonoid biosynthesis. Furthermore, under the comparison of SL vs. CK comparison among the most significantly enriched pathways were those involved in signal transduction and amino acid biosynthesis. The top pathway in enrichment degree was phenylpropanoid biosynthesis under L vs. CK.

### 2.3. WGCNA Analysis

**Figure 4:**
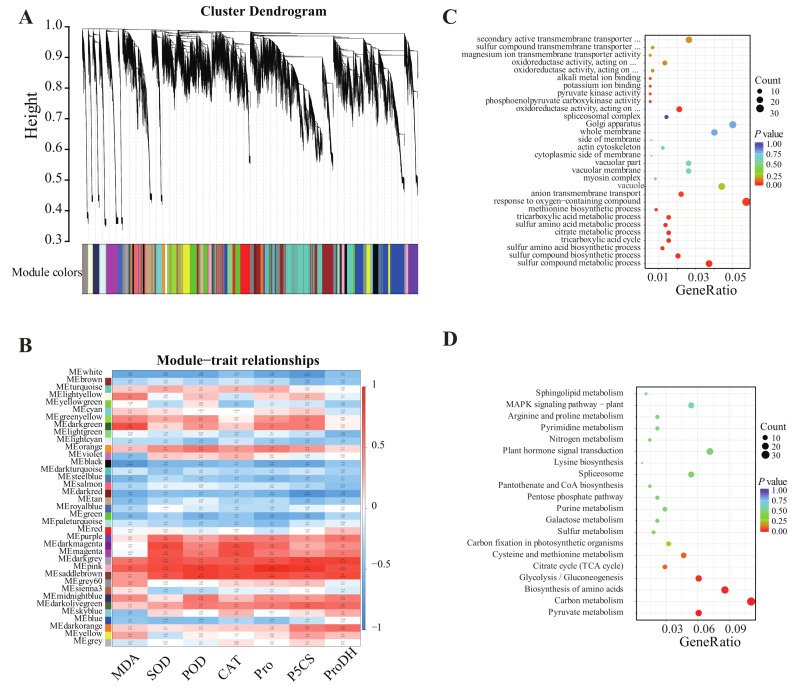
WGCNA module identification, correlation analysis and functional enrichment analysis of DEGs and key module eigengenes. (**A**) Cluster dendrogram of total tomato genes with assigned module colors. (**B**) Correlation of the identified modules with traits under different low R:FR ratio light and/or calcium nitrate stress. MDA: malondialdehyde; SOD: superoxide dismutase; POD: peroxidase; CAT: catalase; Pro: proline; P5CS: Δ 1-pyrrolin-5-carboxylate synthetase; ProDH: proline dehydrogenase. The upper number in each grid represents the correlation coefficient, while the lower is the *p*-value. Red and blue color denotes positive and negative correlation with tomato seedlings’ traits, respectively. (**C**) Gene Ontology (GO) annotation of eigengenes in pink, saddlebrown, and darkgrey modules. (**D**) Kyoto encyclopedia of genes and genomes (KEGG) annotation of eigengenes in pink, saddlebrown, and darkgrey modules.

The WGCNA analysis resulted in 37 distinct modules (Figure 4). Several resistant indexes were identified, including MDA content, SOD activity, POD activity, CAT activity, proline content, PSC5 activity, and ProDH activity (Figure 4B). The results showed that the genes in the three modules of pink, darkgrey and saddlebrown were positively correlated with the resistance index. Among them, the correlation coefficient between the pink module and characters is 0.68–0.97, such as proline (0.97) and P5CS (0.90). Both mesaddlebrown and medarkgrey modules showed significant correlations with ROS indicators (SOD, POD, and CAT) and proline metabolism indicators (proline, P5CS, and ProDH).

The pink module, saddlebrown module, and darkgrey module identified 67 genes, 230 genes, and 71 genes, respectively. The GO and KEGG analyses were performed after merging the three module genes. From Figure 4C, the GO analysis and classification results showed that most (padjust < 0.05) are enriched in the reaction to oxygenates; sulfide synthesis and metabolism, and tricarboxylic acid cycle; and sulfur amino acid biosynthetic and metabolic process during the biochemical process. In the KEGG enrichment analysis, genes were significantly enriched in carbon metabolism, amino acid biosynthesis, pyruvate metabolism, glycolysis/gluconeogenesis, cysteine, and methionine metabolism, as well as hormone signal transduction (Figure 4D).

**Figure 5:**
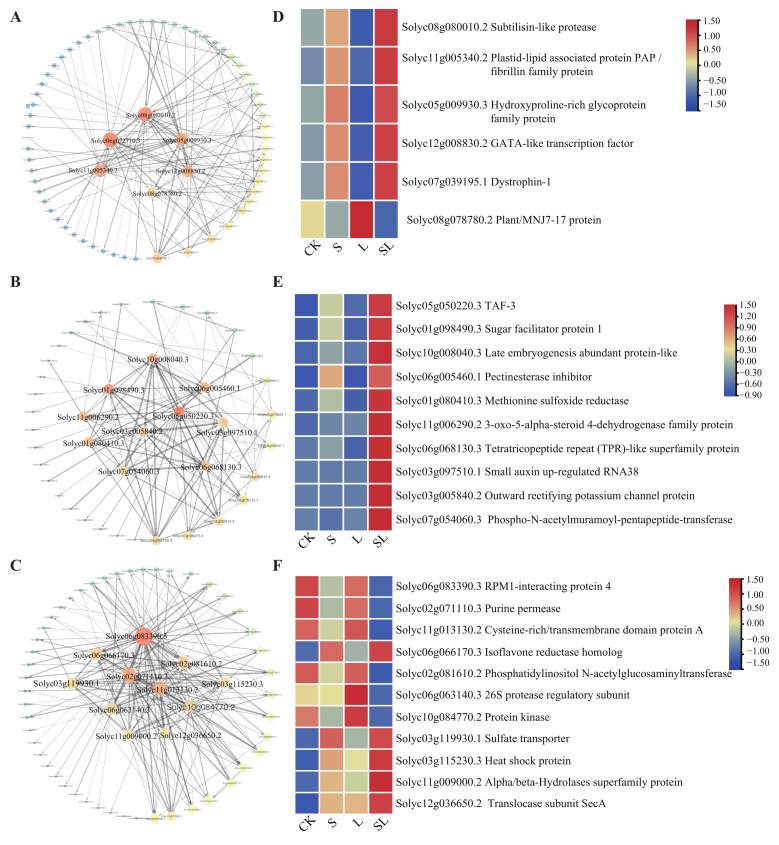
Correlation network of (**A**) pink, (**B**) blue, (**C**) black modules. Genes were selected with the edge weights (**A**) > 0.23, (**B**) > 0.15, (**C**) > 0.12, which were visualized using Cytoscape, respectively. (**D**–**F**) Hub genes in the candidate modules and their genes expression pattern in different treatments.

Three modules with the highest correlation were selected for the gene co-expression network shown in Figure 5A–C. The hub genes were selected based on numbers of edges more than ten. There were 6, 10, and 11 hub genes in the pink module, saddlebrown module, and darkgrey module, respectively (shown in Table S2). Among these genes, there were genes related to stress tolerance in the leaves-specific co-expression modules via WGCNA. One selected gene was Solyc11g005340.2, which encodes plastid–lipid associated protein PAP/fibrillin family protein (FBNs), as shown in Figure 5A. There was also a GATA-like transcription factor (Solyc12g008830.2) in the pink module (Figure 5A). In the saddlebrown modules shown in Figure 5B, one of the selected genes encodes outward rectifying potassium channel protein (Solyc03g005840.2). In the darkgrey module shown in Figure 5C, the top edge numbers of gene (Solyc06g083390.3) encodes RPM1-interacting protein 4. Its homolog RINs were identified in response to immune [15] and salt stress, respectively [16]. Another candidate hub gene was coded by a specific family of sulfate transporter (Solyc03g119930.1) (Figure 5C).

Figure 5D–F shows the expression pattern of the 27 hub genes filtered from the DEGs. In the pink module, the transcription levels of five hub genes were the highest in SL (Figure 5D), including Subtilisin-like protease (Solyc08g080010.2), FBNs (Solyc11g005340.2), Hydroxyproline-rich glycoprotein family protein (Solyc05g009930.3), GATA-like transcription factor (Solyc12g008830.2), and Dystrophin-1 (Solyc07g039195.1). All ten hub genes listed in the saddlebrown module were upregulated by SL (Figure 5E). In the darkgrey module, isoflavone reductase homolog (Solyc06g066170.3), SULTRs (Solyc03g119930.1), heat shock protein (Solyc03g115230.3), alpha/beta-Hydrolases superfamily protein (Solyc11g009000.2), and translocase subunit seca (Solyc12g036650.2) were the hub genes with the upregulated transcription (Figure 5F). However, the gene which encodes RPM1-interacting protein 4 (Solyc06g083390.3) was down-regulated in SL. From Figure S5, we infer that it has many functions in plant stress response, like Arabidopsis sulfate transporters.

### 2.4. RT-qPCR Verification of RNA-Seq Data

**Figure 6:**
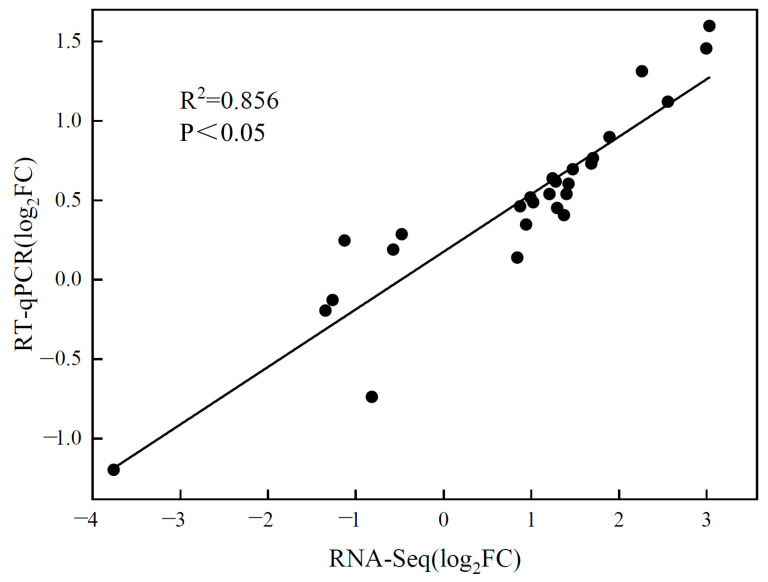
Nine selected genes were validated using the RT-qPCR method comparatively, and linear correlation between RNA-seq and RT-qPCR data was presented with a log2 fold change transformation.

In order to assess the accuracy of RNA-seq data, nine genes from the DEGs were randomly selected for an RT-qPCR. As shown in Figure 6, the correlation coefficient (r^2^) of 0.856 between RNA-seq and RT-qPCR data was significant, indicating that our RNA-seq data were reliable.

## 3. Discussion

The importance of light as a factor of the environment cannot be overstated [17]. In this study, tomato seedlings under calcium nitrate stress grew better and had greater chlorophyll fluorescence when treated with low R:FR ratios. Our previous studies also confirmed this finding [3]. It is suggested that the lower R:FR ratio treatment might reduce the salt-induced inhibition of electron transport and increase tomato seedling salt tolerance.

Under the stress of calcium nitrate, SOD, POD, and CAT activity was significantly increased by low R:FR treatment in tomato seedling leaves, which is similar to the findings of Zhang et al. [18] and Cao et al. [11]. From the RNA-seq analysis, oxidoreductase activity was significantly enriched in the results of the comparison of S vs. CK and SL vs. S (Figure S2). It indicated that calcium nitrate stress can induce the stress response of tomato seedlings to a certain extent and establish a self-defense system to reduce the damage of stress to plants. Low R:FR may enhance the oxidoreductase activity further. At the same time, tomato seedling leaves also showed a decrease in MDA, indicating that low R:FR protected their integrity.

Amino acids were reported to participate directly or indirectly in scavenging free radicals [19] and salt stress responses [20,21]. Under calcium nitrate stress, tomato leaves contained more proline. With low R:FR ratios, proline content and P5CS activity increased under calcium nitrate stress. Osmotic protection and free radical scavenging could be better for salt tolerance [19,22]. From the selected modules of the KEGG enrichment analysis, proline metabolism was also found (Figure 4D). This indicates that the low R:FR ratio may participate in the proline synthesis pathway. Furthermore, amino acid metabolism accounted for a significant proportion of DEGs at salinity stress, involved in proline, methionine, and sulfur amino acids (Figure 4C and Figure S2). Amino acids are considered to be one of the most important and effective organic osmoregulation substances [23]. Sulfur-containing amino acids are the precursors of the endogenous antioxidant glutathione and vitamins, which were found to be important for plant stress tolerance [24].

Salt stress significantly affects the metabolites of stressed plants and enhances some bioactive secondary metabolites in tomato plants. The enhancement of secondary metabolites under salt stress may help to explain the tolerance and sensitivity of tomato to salt stress [25]. The results of GO enrichment and KEGG enrichment show that the effects of calcium nitrate on tomato are mainly concentrated on ion transport and cell components, while the phenylpropanoid biosynthesis pathway is enriched from the comparison of SL vs. S. Phenylpropanes are ubiquitous in plants, and the phenylpropanoid biosynthesis pathway can form most secondary metabolites in plants. There has been evidence that phenylpropanoid contributes to plant growth and development as well as stress responses [24,26].

Fibrillin (FBN) is a plastid–lipid binding protein encoded by a nuclear gene, which originates from photosynthetic prokaryotic cyanobacteria and widely exists in plastids [27,28]. They play an important role in metabolism and signal regulation in plastid differentiation, plant growth and development, and resistance to abiotic and biotic stresses [28]. Some evidence shows that fibroin not only participates in oxidative stress tolerance [29,30], but also in hormone signal transduction, especially jasmonic acid and ABA signal transduction [29,30,31]. Sulfate transporters (SULTRs) were another candidate hub gene in the darkgrey module, which are coded by a specific family of sulfate transporter genes. SULTRs proteins are widely involved in plant stress response processes, such as heavy metal, low-temperature, drought, salt and other abiotic stresses [32,33,34]. In this study, the hub genes (Solyc11g005340.2 and Solyc03g119930.1), encoding FBN and SULTRs were upregulated by the low R:FR ratio under calcium nitrate treatment (Figure 5D–F), which could be functionally characterized using reverse genetic experiments. In addition, Rin4 is localized at the plasma membrane, and it can regulate cell balance and reactive oxygen species balance by activating plasma membrane H ^+^- ATPase, thus making plants respond to salt stress. The heterologous expression of the PeRIN gene of *Populus euphratica* improved the salt tolerance of *Arabidopsis thaliana* [35]. However, Solyc06g083390.3, the hub gene with the highest edge number (45 edges)—which was down-regulated by the low R:FR ratio under excess nitrate stress—is still unknown in tomato.

GATA transcription factors are ubiquitous in eukaryotes and play an important role in some important biological processes, such as plant light response regulation, cytokinin response, abiotic stress resistance, chlorophyll synthesis, and carbon and nitrogen metabolism. By means of bioinformatics, 30 tomato GATA transcription factor family members were identified and obtained [36]. Am zfpg belongs to the GATA family and has high conservation. The results showed that the expression of am zfpg gene increased under low temperature and drought stress, which could predict that the expression of am zfpg gene might be positively correlated with drought and low-temperature stress [37].

## 4. Materials and Methods

### 4.1. Plant Materials and Treatment Design

In this study, tomato seedlings (*Solanum lycopersicum* L. cv. Jin peng NO.1) were used as the experimental material. The study was conducted at the College of Horticulture of Sichuan Agricultural University. Tomato seeds were selected, soaked, and planted in a plant factory. The experiment was maintained at 25–27 °C during the day and 17–19 °C during the night (12 h light/12 h dark). The relative humidity was 70 ± 2% and the light intensity was 200 ± 25 μmol·m^−2^s^−1^. After that, the tomato seedlings with the third true leaf expanded were transplanted into the a matrix culture container (15 × 13 cm) and approximately 1/2 Hoagland’s solution applied every four days.

In the plant factory, the R:FR ratio can be adjusted by combining the white and far-red LED lamps according to different quantities, on the premise that the photosynthetic active radiation in the environment is unchanged. The light intensity was measured by a spectrometer (OHSP350P, China), and the R:FR ratio was calculated according to the method of Zhou et al. [3]. A total of 4 treatments were set up, as follows in Table 1:

Five days after treatment, the fully expanded functional leaves from the selected nine plants (3 samples per sample, 3 samples per treatment) were frozen and stored at −80 °C in a refrigerator.

### 4.2. Determination of Morphological Indexes

The measurement of plant height and root length was carried out using a ruler after the absorption of water with absorbent paper. The dry weight was determined by the method of Zhou et al. [3].

#### 4.2.1. Determination of Proline Metabolism, Malondialdehyde Content, and Antioxidant Enzyme Activity

Using ninhydrin colorimetry, we calculated the proline content in the leaves [38]. The activity of P5CS (1-pyrrolin-5-carboxylate synthetase) and ProDH was measured using ELISA. The MDA content was determined according to Cakmak and Horst [39]. The SOD activity was determined according to Beauchamp and Fridovich [40]. The POD activity was determined according to Kraus and Fletcher [41]. The CAT activity was determined based on Chance and Maehly [42]. According to Zhou et al. [43], the histochemical staining method of hydrogen peroxide was determined.

#### 4.2.2. Chlorophyll Fluorescence Parameters Determination

Five days after treatment, the PAM-2500 chlorophyll fluorescence meter (Walz, Germany) was used to measure the chlorophyll fluorescence parameters. Based on the above indicators, the maximum quantum efficiency (Fv/Fm) of PSII photochemistry, the effective photon yield (Fv’/Fm’) of PSII, and the actual electron transport efficiency of PSII (Y(II)) and non-photochemical quenching (NPQ) were calculated [44].

### 4.3. Transcriptome Analysis

After the tomato leaf sample was ground into powder with liquid nitrogen, the total RNA was extracted with Trizol extraction solution (Invitrogen, Carlsbad, CA, USA). The total amount of RNA was detected with the Agilent 2100 Bioanalyzer (Thermo Scientific, Waltham, MA, USA). RNA SEQ library construction and sequencing were completed by Nuohe Zhiyuan Biotech Co., Ltd. (Beijing, China). High-throughput sequencing was performed on the Illumina HiSeq 2500 technology sequencing platform [45].

The raw reads data generated were filtered to remove the linker sequence and low-quality reads. Hisat2 v2.0.5 was used to compare the clean reads with the reference genome. The differential expression between the two comparison combinations was analyzed using DESeq2 software (1.20.0). In screening, *p*-value < 0.05 and |log2FoldChange| > 0 were used as the screening criteria. By using cluster Profiler (3.4.4), we analyzed Gene Ontology (GO) enrichment and the Kyoto Encyclopedia of Genes and Genomes (KEGG) enrichment.

### 4.4. Weighted Gene Co-Expression Network Analysis

The co-expression network and hub genes were constructed using WGCNA [46], The visualization of networks was constructed through the Cytoscape3.7.2 software.

### 4.5. RT-qPCR Validation

In this study, an RT-qPCR was conducted using the method of Zhou et al. [3]. The primer sequences are shown in Table S1.

### 4.6. Data Processing and Analysis

Microsoft Excel 2016 software was used to sort out the data, and SAS was used to analyze the variance of the data. Duncan’s new compound extreme difference method was used, and GraphPad prism 8 was used for mapping.

## 5. Conclusions

In this study, a weighted gene co-expression network analysis (WGCNA) and related transcriptome analysis provided evidence that a low R:FR ratio improves plant salt tolerance, and we found the active involvement of plant hormone signal transduction, amino acid metabolic and phenylpropanoid biosynthesis, reaction to oxygenates, sulfide synthesis and metabolism, and oxidoreductase activity. In addition, we identified important novel hub genes encoding proteins including FBNs, SULTRs, and GATA-like transcription factor, which may play a vital role in low R:FR ratio light-induced salt responses. Our findings offer a new perspective of the mechanism and environmental implication behind low R:FR ratio light-modulated tomato saline tolerance, and they could be of particularly great value to future research on the hub genes involved in salt tolerance.

## Figures and Tables

**Table 1:** Nutrient solution and light source of each treatment.

Treatment	Nutrient Solution	Light Source
CK	1/2 Hoagland’s solution	white LED (R:FR = 4.2)
L	1/2 Hoagland’s solution	white LED + far-red LED (R:FR = 0.7)
S	1/2 Hoagland’s solution Including 160 mM of Ca(NO_3_)_2_	white LED (R:FR = 4.2)
SL	1/2 Hoagland’s solution Including 160 mM of Ca(NO_3_)_2_	white LED + far-red LED (R:FR = 0.7)

## Data Availability

Data available on request due to restrictions, e.g., privacy or ethical. The data presented in this study are available on request from the corresponding author.

## Supplementary Materials

The following supporting information can be downloaded at: https://www.mdpi.com/article/10.3390/ijms24043738/s1.
