# Supplementary material for: Transcriptome Mechanisms of Tomato Seedlings Induced by Low-Red to Far-Red Light Ratio under Calcium Nitrate Stress
Source: Int J Mol Sci. 2023 Feb 13;24(4):3738. doi: 10.3390/ijms24043738 (PMC9963801; doi:10.3390/ijms24043738)
Supplement: Supplementary file 1 [file ijms-24-03738-s001.zip › Figure Captions.pdf]

Figure Captions

Figure S1. Heat map representation of DEGs.

Figure S2. The Gene Ontology (GO) enrichment analysis of DEGs in the comparisons of S vs CK (A), SL vs. S (B), SL vs. CK (C), and L vs. CK (D). The dot size and the dot color indicate the number of DEGs of the pathway and q value, respectively.

Figure S3. KEGG pathway enrichment analysis of the annotated DEGs in the comparisons of S vs CK, SL vs. S, SL vs. CK, and L vs. CK.

Figure S4. (A) and (B) Soft threshold in scale independence and mean connectivity，respectively.

Figure S5. Evolutionary relationships of sulfate transporters (SULTRs) polypeptides from *Arabidopsis*, *Zea mays* L., rice (*Oryza sativa* L.), and tomato (*Solanum lycopersicum* L.). The MEGA4 software package (http://www.megasoftware.net/mega4/mega.html) was used for phylogenetic analysis. The scale bar indicates the number of amino acid substitution per site. The proposed SULTR protein designations for a tomato sulfate transporter were shown in red.
