# Supplementary material for: Transcriptome Mechanisms of Tomato Seedlings Induced by Low-Red to Far-Red Light Ratio under Calcium Nitrate Stress
Source: Int J Mol Sci. 2023 Feb 13;24(4):3738. doi: 10.3390/ijms24043738 (PMC9963801; doi:10.3390/ijms24043738)
Supplement: Supplementary file 1 [file ijms-24-03738-s001.zip › Tables S1-S3.pdf]

Supplemental information

Supplementary Table S1

Primer sequences used for quantitative Real-Time PCR

Gene name	Primer	Primer sequences (5'-3')
Solyc01g102300	Forward	GGGATGGGATTTGGGTTGGGTATG
	Reverse	GATAAGCGGTGGAAGCAGGAATAGG
Solyc08g076970	Forward	GGGAACTTGTGGTAACTGGAAAGGG
	Reverse	AGCGGGTTTGAATTTCTTTGAGTGC
Solyc11g069430	Forward	GGTGGTGTTAATGTCATGGCAGGAG
	Reverse	TTTAGGGTCGGTGGCGGAGAAG
Solyc11g007690	Forward	TGCCTTGAATCGGGAATGTCTGTG
	Reverse	TAGTTCAGGACCACCAGTGTCTAGC
Solyc06g071000	Forward	GGTCCTGTCCTTACGGTTACAACTG
	Reverse	AGGCTTCACCACCAAAGAGCAATC
Solyc02g093720	Forward	ATGTAGAGGAGGCTGAGAGGTATGC
	Reverse	CGGGGTTTTGTTGATTGGCTTGTTC
Solyc10g081840	Forward	CTTACTTAAACCGCGCAAGCCATTC
	Reverse	CCATCCGTCTTCCTCGTGTTCAAG
Solyc08g044260	Forward	TGGCTTCAAAACCAGGGATTCTCAC
	Reverse	TCCCACAAATGGTGCCAAAACAATG
Solyc02g083130	Forward	CTCAAGCCACCAGAAGAGCGTATAG
	Reverse	GGCAACTTCCGATTCTCCGTCATC
actin	Forward	TGGTCGGAATGGGACAGAAG
	Reverse	CTCAGTCAGGAGAACAGGGT

Supplementary Table S2

The hub genes in the pink module, saddlebrown module and darkgrey module

Gene name	Description	Degree
**Pink module**		
Solyc08g080010.2	Subtilisin-like protease	20
Solyc11g005340.2	Plastid-lipid associated protein PAP / fibrillin family protein	17
Solyc05g009930.3	Hydroxyproline-rich glycoprotein family protein	16
Solyc12g008830.2	GATA-like transcription factor	14
Solyc07g039195.1	Dystrophin-1	10
Solyc08g078780.2	LOW QUALITY:Plant/MNJ7-17 protein, putative	10
**Saddlebrown module**		
Solyc05g050220.3	TAF-3	19
Solyc01g098490.3	Sugar facilitator protein 1	17
Solyc10g008040.3	Late embryogenesis abundant protein-like	15
Solyc06g005460.1	LOW QUALITY:Pectinesterase inhibitor	13
Solyc01g080410.3	Methionine sulfoxide reductase, putative	12
Solyc11g006290.2	3-oxo-5-alpha-steroid 4-dehydrogenase family protein	12
Solyc06g068130.3	Tetratricopeptide repeat (TPR)-like superfamily protein	12
Solyc03g097510.1	Small auxin up-regulated RNA38	11
Solyc03g005840.2	Outward rectifying potassium channel protein	11
Solyc07g054060.3	Phospho-N-acetylmuramoyl-pentapeptide-transferase, putative	10
**Darkgrey module**		
Solyc06g083390.3	RPM1-interacting protein 4	45
Solyc02g071110.3	Purine permease	31
Solyc11g013130.2	Cysteine-rich/transmembrane domain protein A	28
Solyc06g066170.3	Isoflavone reductase homolog	19
Solyc02g081610.2	Phosphatidylinositol N-acetylglucosaminyltransferase	19
Solyc06g063140.3	26S protease regulatory subunit, putative	18
Solyc10g084770.2	Protein kinase	17
Solyc03g119930.1	Sulfate transporter, putative	16
Solyc03g115230.3	Solanum lycopersicum heat shock protein	16
Solyc11g009000.2	Alpha/beta-Hydrolases superfamily protein	12
Solyc12g036650.2	translocase subunit seca	11

Supplementary Table S3

Amino acid sequence information on sulfate transporters

Species	Proposed SULTRs protein	Locus tag
Solanum lycopersicum	SULTR	Solyc03g119930.1
Arabidopsis thaliana	AtSULTR3;1	AT3G51895
Arabidopsis thaliana	AtSULTR3;2	AT4G02700
Arabidopsis thaliana	AtSULTR3;3	AT1G23090
Arabidopsis thaliana	AtSULTR3;4	AT3G15990
Arabidopsis thaliana	AtSULTR3;5	AT5G19600
Arabidopsis thaliana	AtSULTR4;1	AT5G13550
Arabidopsis thaliana	AtSULTR4;2	AT3G12520
Zea mays	ZmSULTR2;1	ZEAMMB73_Zm00001d028164
Zea mays	ZmSULTR3;3	ZEAMMB73_Zm00001d002038
Zea mays	ZmSULTR3;4	ZEAMMB73_Zm00001d000204
Zea mays	ZmSULTR3;5	ZEAMMB73_Zm00001d043614
Oryza sativa	OsSULTR1;1	AF493790
Oryza sativa	OsSULTR1;2	ABF94450
Oryza sativa	OsSULTR1;3	AF493790
Oryza sativa	OsSULTR2;1	AAN59769
Oryza sativa	OsSULTR2;2	AAN59770
Oryza sativa	OSSULTR3;1	NP_921514
Oryza sativa	OsSULTR3;2	AAN06871
Oryza sativa	OsSULTR3;3	AK104831
Oryza sativa	OsSULTR3;4	BAD68396
Oryza sativa	OsSULTR4;1	AF493793
