# Supplementary figures and images for: Transcriptome Mechanisms of Tomato Seedlings Induced by Low-Red to Far-Red Light Ratio under Calcium Nitrate Stress
Source: Int J Mol Sci. 2023 Feb 13;24(4):3738. doi: 10.3390/ijms24043738 (PMC9963801; doi:10.3390/ijms24043738)

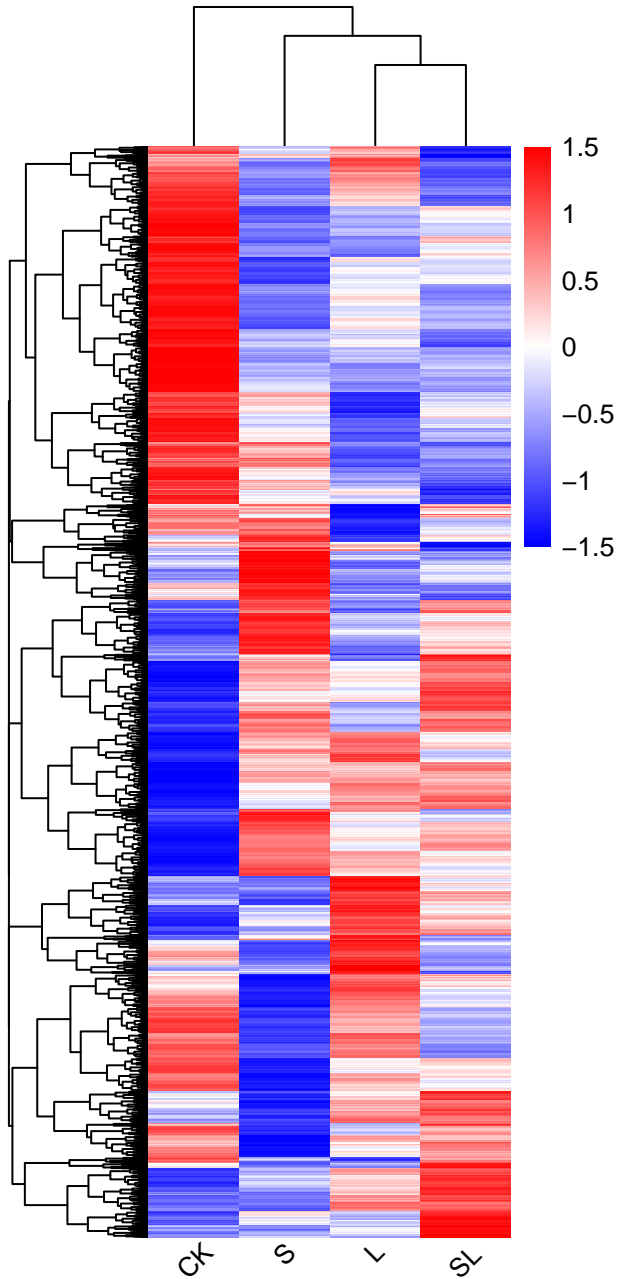

Supplement: Supplementary file 1 [file ijms-24-03738-s001.zip › FigureS1.pdf]

Description

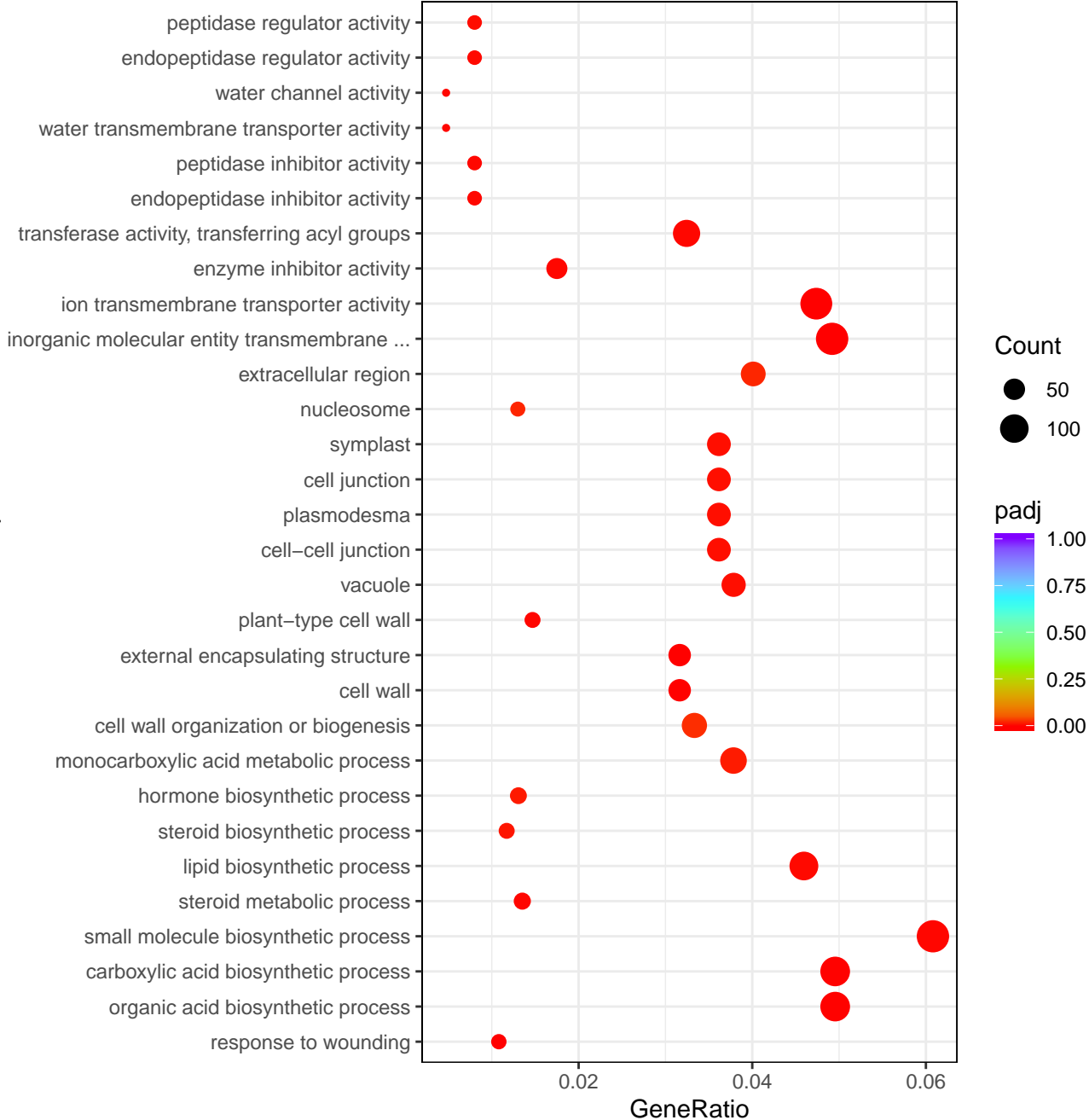

Supplement: Supplementary file 1 [file ijms-24-03738-s001.zip › FigureS2A.pdf]

Description

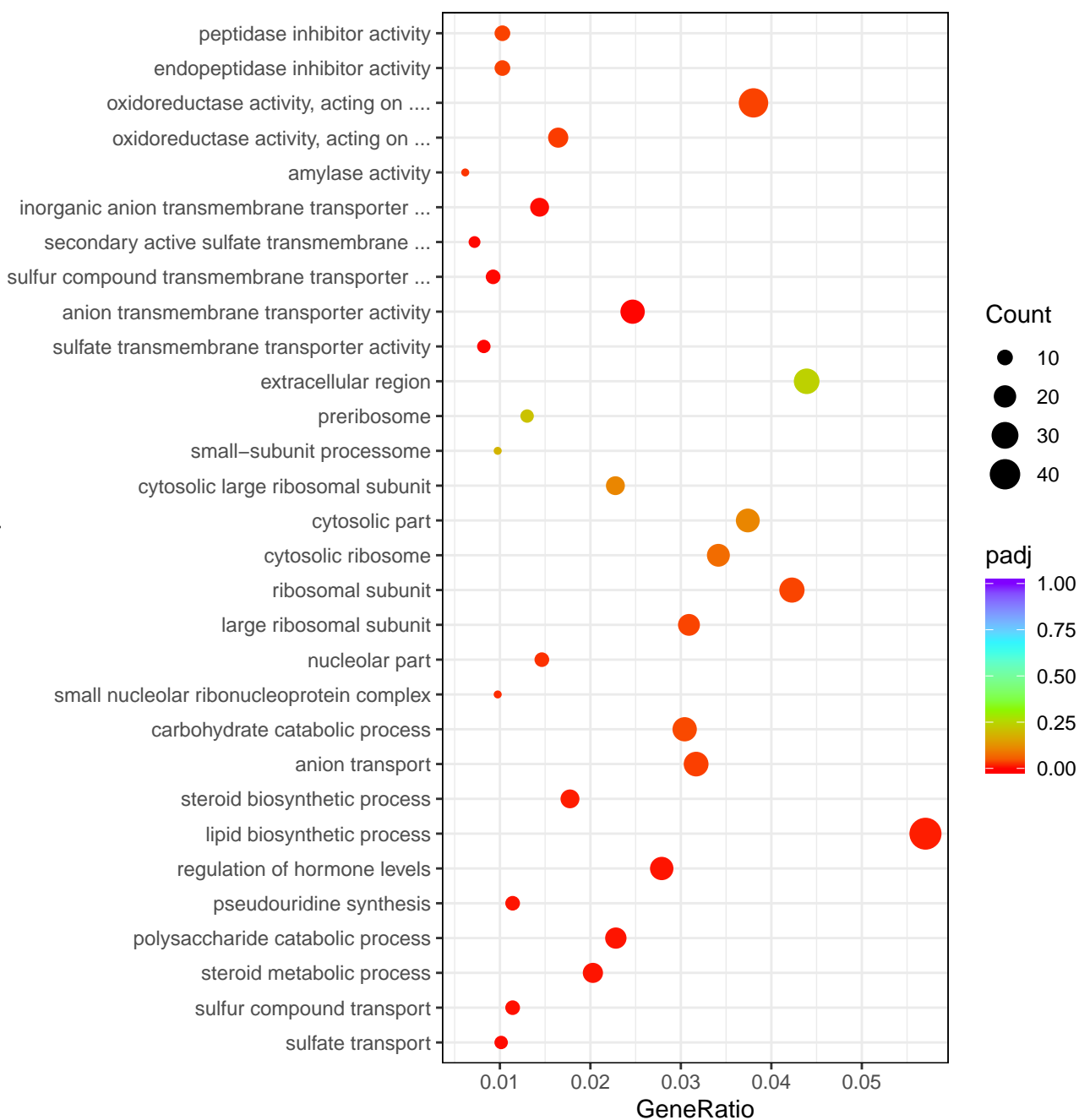

Supplement: Supplementary file 1 [file ijms-24-03738-s001.zip › FigureS2B.pdf]

Description

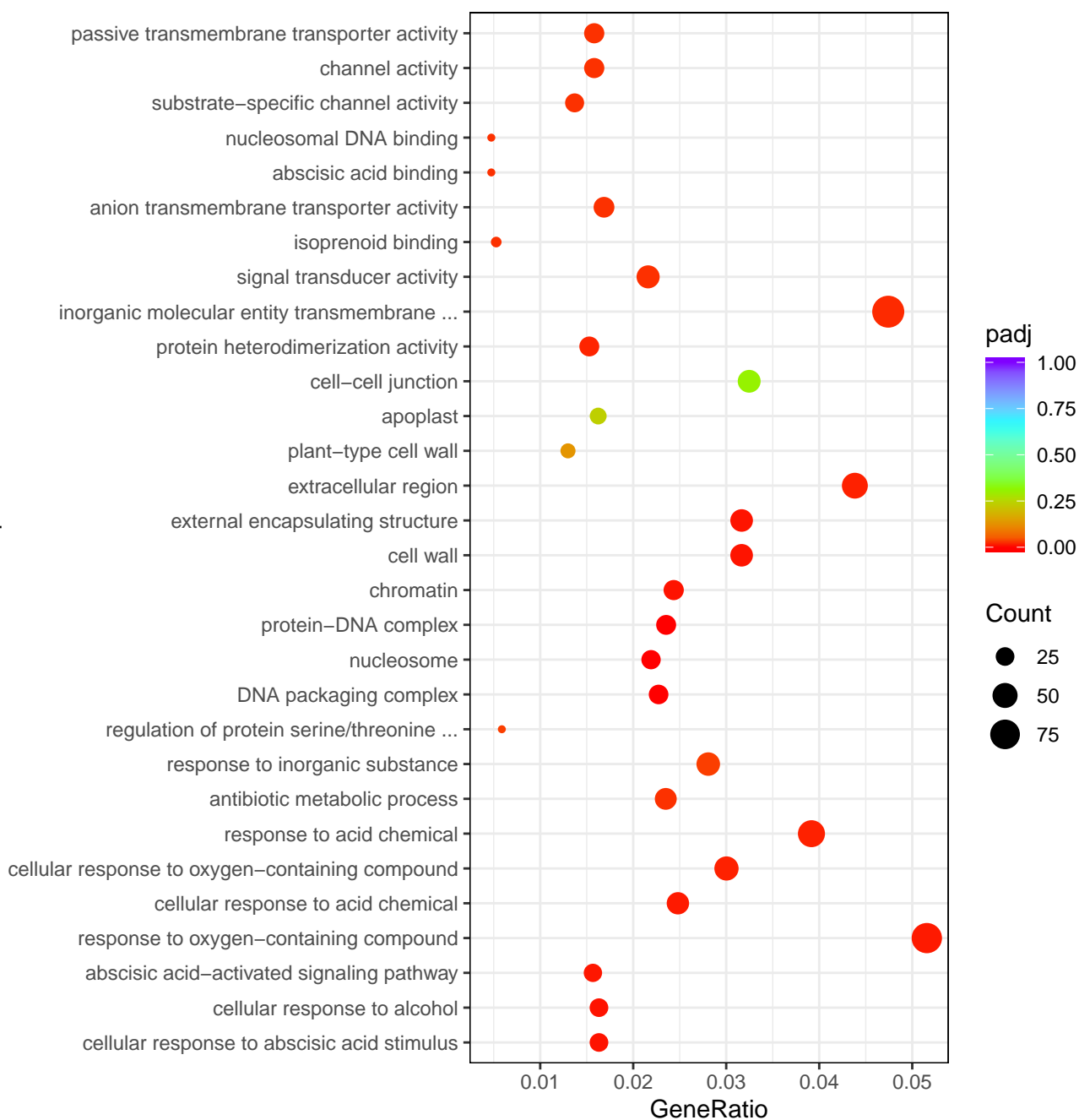

Supplement: Supplementary file 1 [file ijms-24-03738-s001.zip › FigureS2C.pdf]

Description

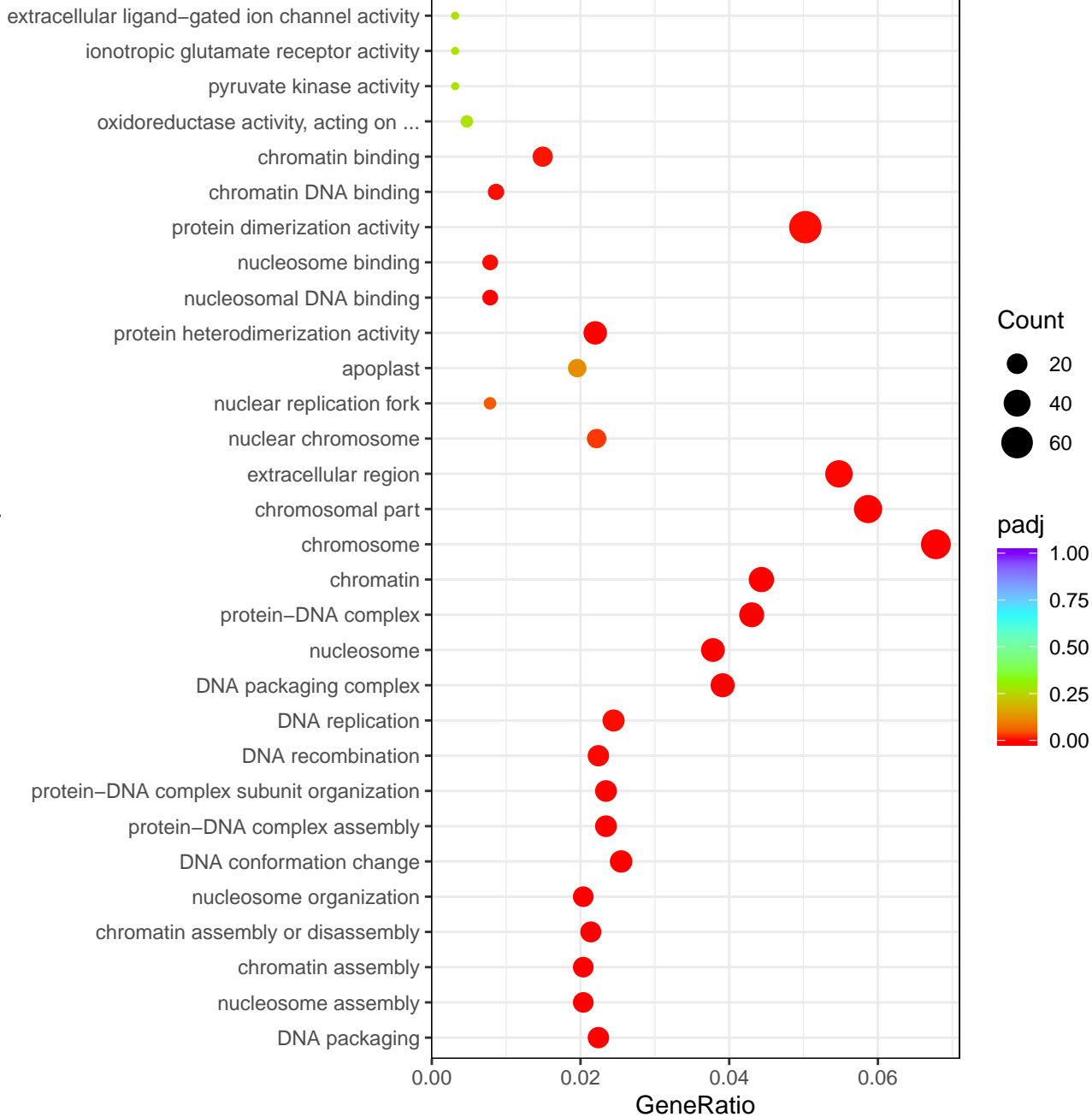

Supplement: Supplementary file 1 [file ijms-24-03738-s001.zip › FigureS2D.pdf]

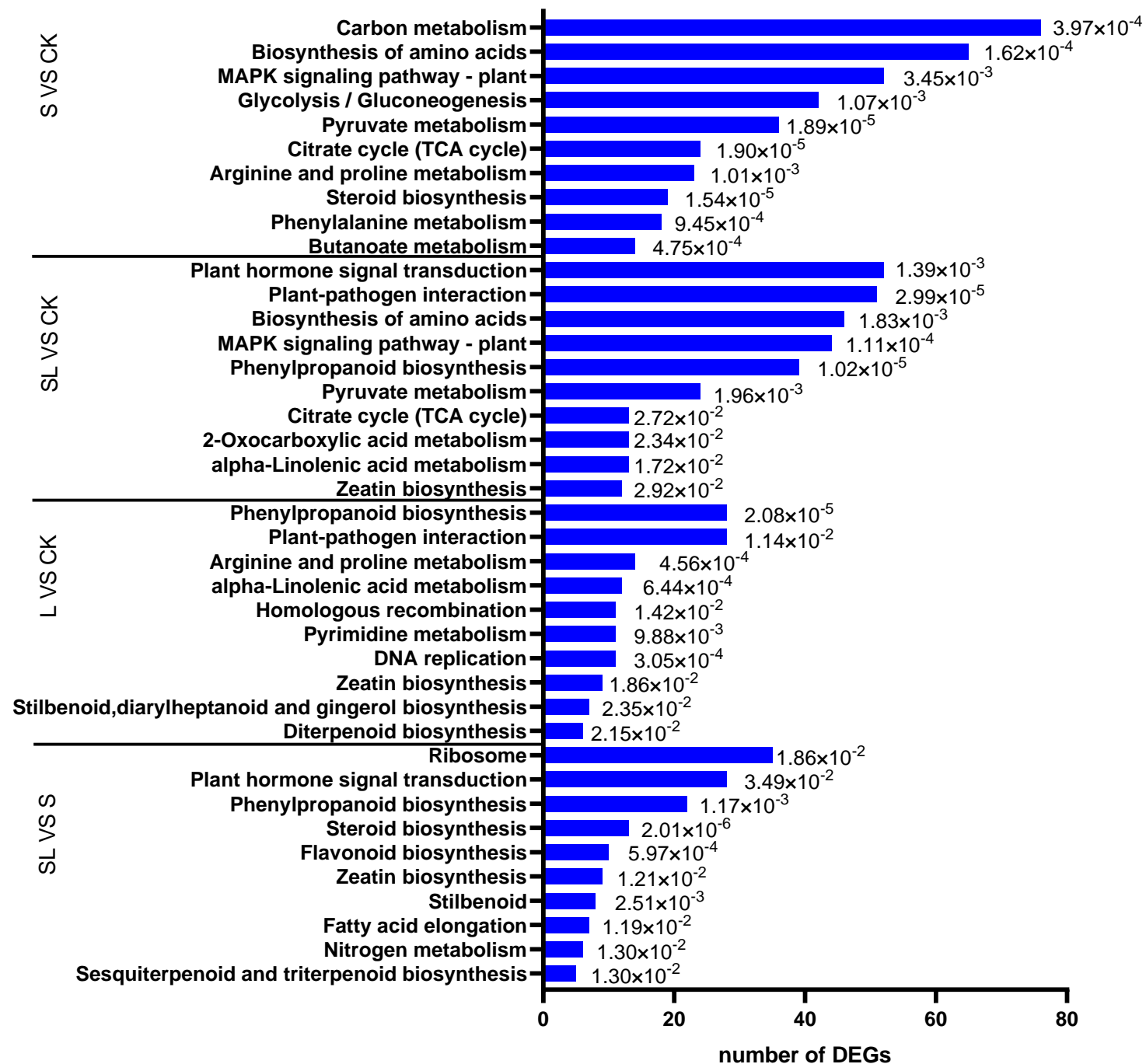

Supplement: Supplementary file 1 [file ijms-24-03738-s001.zip › FigureS3.pdf]

**A**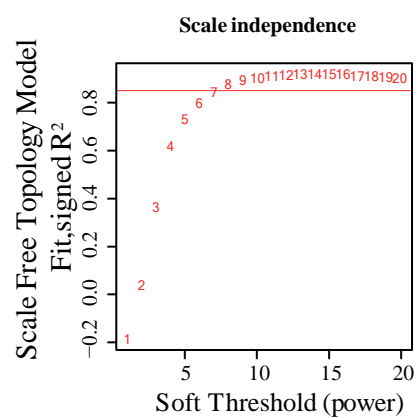**B**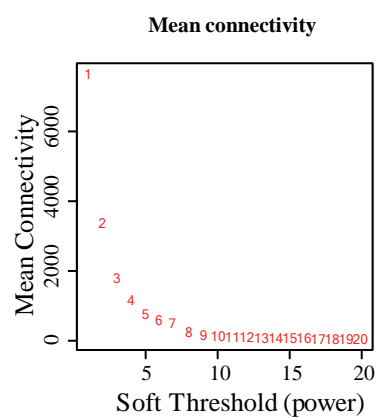

Supplement: Supplementary file 1 [file ijms-24-03738-s001.zip › FigureS4.pdf]

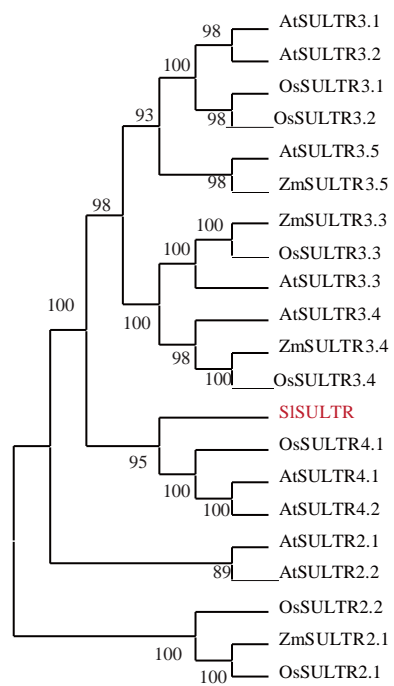

Supplement: Supplementary file 1 [file ijms-24-03738-s001.zip › FigureS5.pdf]
